# Benchmarking Foot Trajectory Estimation Methods for Mobile Gait Analysis

**DOI:** 10.3390/s17091940

**Published:** 2017-08-23

**Authors:** Julius Hannink, Malte Ollenschläger, Felix Kluge, Nils Roth, Jochen Klucken, Bjoern M. Eskofier

**Affiliations:** 1 Machine Learning and Data Analytics Lab, Department of Computer Science, Friedrich-Alexander University Erlangen-Nürnberg (FAU), 91054 Erlangen, Germany; malte.ollenschlaeger@gmail.com (M.O.); felix.kluge@fau.de (F.K.); nils.roth@fau.de (N.R.); bjoern.eskofier@fau.de (B.M.E.); 2 Department of Molecular Neurology, University Hospital Erlangen, Friedrich-Alexander University Erlangen-Nürnberg (FAU), 91054 Erlangen, Germany; jochen.klucken@uk-erlangen.de

**Keywords:** wearable sensors, human gait, clinical gait analysis, benchmark dataset, orientation estimation, double integration

## Abstract

Mobile gait analysis systems based on inertial sensing on the shoe are applied in a wide range of applications. Especially for medical applications, they can give new insights into motor impairment in, e.g., neurodegenerative disease and help objectify patient assessment. One key component in these systems is the reconstruction of the foot trajectories from inertial data. In literature, various methods for this task have been proposed. However, performance is evaluated on a variety of datasets due to the lack of large, generally accepted benchmark datasets. This hinders a fair comparison of methods. In this work, we implement three orientation estimation and three double integration schemes for use in a foot trajectory estimation pipeline. All methods are drawn from literature and evaluated against a marker-based motion capture reference. We provide a fair comparison on the same dataset consisting of 735 strides from 16 healthy subjects. As a result, the implemented methods are ranked and we identify the most suitable processing pipeline for foot trajectory estimation in the context of mobile gait analysis.

## 1. Introduction

Mobile gait analysis systems are based on Inertial Measurement Units (IMUs) that are mounted on the subject and allow an objective assessment of gait [1]. As such, they are used in a variety of applications ranging from the medical domain to consumer electronics. One prominent example is the use of these systems in neurodegenerative diseases such as Huntington’s, Alzheimer’s and Parkinson’s Disease (PD). PD patients, for example, exhibit a variety of symptoms that, amongst others, affect gait and reduce their quality of life [2]. A few examples of gait disturbances observed in PD patients range from a reduced stride length over shuffling gait to episodes of complete gait freezing [3]. This change in gait pattern is observed by physicians in order to stage PD severity and provide appropriate treatment. The clinical gold standard for disease progression and motor function is the Movement Disorder Society’s revision of the Unified Parkinson’s Disease Rating Scale (MDS-UPDRS) [4]. However, only one item on this scale directly concerns gait and is assessed by means of visual observation. This represents a subjective and incomplete snapshot of the patient’s gait performance since visual gait assessment is long known to be unreliable [5]. Furthermore, patients as well as physicians might be influenced by the clinical assessment paradigm [6]. Addressing this problem would require unobtrusive means of measurement and less direct assessment schemes. Therefore, an objective and mobile measurement system is desirable for clinical gait analysis.

Unlike lab-based systems such as pressure carpets or motion capture systems, body-worn IMUs need little subject preparation prior to assessment and are therefore practical in a clinical environment [1]. Moreover, such mobile systems could be used for gait analysis in the patient’s home environment due to the mobility/wearability of the sensing technology. This would allow a more continuous assessment of disease progression as opposed to snapshot visits in specialist centers which is the current state-of-the-art. Additionally, an automated assessment of gait impairment and delivery of clinically meaningful summary measures would help to document and evaluate disease progression and therapeutic interventions [7].

One central component in these sensor-based, mobile gait analysis systems is the reconstruction of the foot trajectory from IMU data captured during, e.g., standardised clinical walking trials. This reconstruction is necessary since the parameters of interest, i.e., positional/orientation information, cannot be measured directly due to the physical constraints in the wearable sensing scenario. No external base stations, as in a motion capture setting, are available that would allow positional tracking.

**Figure 1:**
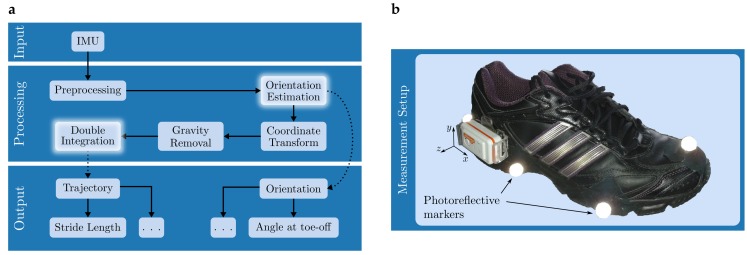
(**a**) Conceptual workflow for estimating foot trajectory and orientation in the context of mobile gait analysis with Inertial Measurement Units (IMUs). Incoming data is processed on a stride-by-stride level in order to provide relevant features as, e.g., stride length or foot orientation at certain key events during the gait cycle. This methodological benchmarking study is specifically concerned with the highlighted blocks; (**b**) An IMU is placed on the lateral side of the shoe below the ankle. The coordinate system of measurement is aligned as indicated above. Additionally, photoreflective markers are attached in order to provide reference data regarding foot position and orientation.

Conceptionally, the processing pipeline for the reconstruction task can be described as shown in  Figure 1a. Given IMU data as input, position and orientation are computed on a stride-by-stride basis in order to provide a parametrisation of the gait pattern. This parametrisation aims at capturing relevant information in a low dimensional space compared to the complete trajectory data (position and foot orientation for every instant). In the clinical use case, these summary descriptions of gait performance are the main output of sensor-based mobile gait analysis systems that ensure practicality [7]. Other use-cases of such systems could involve visualisations of the foot trajectory for, e.g., tele-monitoring concepts or patient feedback mechanisms.

While some steps from the processing layer in  Figure 1a are rather generic, others like orientation estimation or double integration might be based on a variety of methods described in literature. Regarding orientation estimation, suitable methods range from gyroscope integration as proposed by Sabatini [8] to more advanced methods that incorporate accelerometer data for orientation sensing. Those advanced methods include complementary filters (CFs) as proposed by Madgwick et al. [9] or Euston et al. [10]. Double integration methods have to satisfy a variety of boundary conditions. One important boundary condition is the the zero-velocity assumption that requires the foot to be stationary at the start and end of a stride. Based on this, integration is re-initialized by controlling initial and final values of computed integrals. In literature, the zero velocity assumption is addressed in quite diverse ways. One solution is direct integration followed by the identification and removal of integration drift as implemented by Sabatini [8], Mariani et al. [11], Rebula et al. [12], Rampp et al. [13] or Kitagawa and Ogihara [14]. Other approaches, however, try to incorporate these cyclic boundary conditions in less direct ways. Zok et al. [15] proposed an integration scheme that fuses a regular with a time-reversed integral in order to satisfy the boundary condition. This is used by Trojaniello et al. [16] in the context of mobile gait analysis. Other approaches include the recently proposed analytic integration scheme described by Sabatini et al. [17] that is based on Fourier decomposition.

In summary, there is large variation in potential processing pipelines for foot trajectory estimation in mobile gait analysis and no clear objective which configuration to choose for a specific application. Moreover, a lack of publicly available and generally excepted benchmark datasets in this field results in performance evaluations reported on a variety of datasets that are not comparable [18]. A unified evaluation of different processing pipelines for foot trajectory estimation on the same dataset is therefore needed to complement existing literature. We already expressed this need in prior work [18,19] and address it with this manuscript. We specifically look at orientation estimation and double integration methods in the context of mobile gait analysis. We implement and test three different methods drawn from literature for each of the two processing steps with the aim to identify the most suitable processing pipeline for foot trajectory estimation.

## 2. Dataset

The dataset used for this work was acquired previously by Kanzler et al. [20]. It consists of IMU data captured at the feet and annotated with time-synchronised position measurements obtained from a VICON motion capture system. In total, 20 healthy subjects between 16 and 80 years of age were asked to complete 15 walking trials of 10 m each. In order to obtain large variation in stride length and velocity, subjects were asked to modulate both parameters to reach low, normal and high ranges. Additionally, low and high range regimes were coupled to get data on long strides at low gait velocity and vice versa. Each condition was repeated three times. Although IMU data was captured with two different sensor systems, we only focus on the subset acquired with the Shimmer2R sensor platform [21] consisting of a 3D-accelerometer (range ±6g) and a 3D-gyroscope (range $\pm \;500^{\circ }/s$). Here, the total amount of data summed to 735 strides from 16 subjects due to errors during data acquisition like partial failure of one of the two systems. All subjects gave written, informed consent prior to data acquisition and the study was conducted in accordance with the Declaration of Helsinki.

Figure 1b shows the inertial measurement setup used during data collection. A Shimmer2R IMU was attached to the lateral side of the shoe below the ankle and aligned with the functional axes of the foot. All subjects wore the same shoe model (adidas Duramo 3) since an influence of footwear on gait patterns is known from literature [22]. IMU data was recorded at a sampling rate of 102.4 Hz and streamed via bluetooth to a mobile device for storage. The reference VICON motion capture system consisted of 16 VICON T-Series cameras that were partly mounted on a scaffold at three meters height or on tripods on the ground such that a volume of 5×3×2 m could be captured. With this system, data was captured at 200 Hz and later resampled to the IMU’s sampling rate. In order to obtain direct measurements of position, photoreflective markers were positioned on the lateral and medial calcaneus, the lower part of the calcaneal tendon, the first and the fifth metatarsal head and the distal phalanx of the first toe. Calibration of the VICON system was done with a calibration wand and synchronisation between both measurement systems was accomplished with a wireless trigger system as described by Kugler et al. [23]. The foot position and orientation at mid-stance defined the coordinate system in which position measurements were obtained from the VICON for the current stride in order to align measurement frames between both systems.

## 3. Methods

The previously recorded IMU data now serves as input for the processing layer in  Figure 1a. The methods concerning *Preprocessing*, *Coordinate Transform* and *Gravity Removal* are fixed within this work. For *Orientation Estimation* as well as *Double Integration*, three different approaches are evaluated. Orientations are represented in terms of quaternions and the appendix gives an overview on relevant mathematics. In the following, quaternions will describe sensor-to-world rotations and we drop any prescript indicating this for reasons of readability.

### 3.1. Preprocessing

In the context of gait analysis, preprocessing consists of sensor calibration, stride segmentation and gait event detection. Sensor calibration is done according to Ferraris et al. [24]. In our case, stride segmentation is already supplied by the dataset. In the final application, however, it could be achieved using dynamic time warping with a stride template as explained by Barth et al. [25]. For gait event detection, mid-stance (MS) events are estimated according to Rampp et al. [13]. Briefly, MS is defined as the point of minimal energy in the gyroscope signal of a stride. Two successive mid-stances at $t_{ms}$ and $t_{ms+1}$ are then used as the underlying stride definition that allows re-initialization of orientation estimation and double integration after each stride due to the assumption that the foot is stationary at these timepoints (zero-velocity assumption).

### 3.2. Orientation Estimation

Estimation of initial sensor orientation during MS does not vary between methods. It is limited to inclination estimation since heading information is not available from accelerometer or gyroscope data. The inclination is estimated using the accelerometer signal $a_{s}\left(t\right)$ in the sensor frame, which is assumed to contain no movement component at MS. Therefore, rotation angles around the transversal (*z*) and anterior-posterior axis (*x*) can be calculated as:

(1) $$\begin{array}{cc} \tan(\alpha_{z})=-\frac{a_{x}}{a_{y}} & \tan(\alpha_{x})=\frac{a_{z}}{\sqrt{a_{x}^{2}+a_{z}^{2}}} \end{array}$$

Using Equations (A3) and (A4), two rotation quaternions $q_{z}$ and $q_{x}$ are defined and their quaternion product ⊗ yields the initial quaternion:

(2) $$\begin{array}{c} q\left(t_{ms}\right)=q_{z}⊗q_{x} \end{array}$$

**Figure 2:**
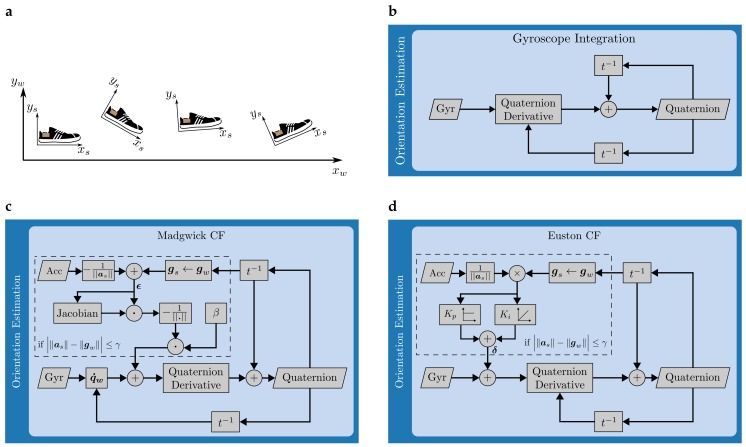
(**a**) Coordinate systems in the sagittal plane. Subscripts denote the sensor- and world-coordinate system; (**b**–**d**) Block diagrams for the three orientation estimation techniques implemented within this work.

Due to changes of the foot orientation as shown in  Figure 2a, the initial quaternion needs to be updated at each sample within the stride ($t_{ms}<t\leq t_{ms+1}$). This update is performed differently for each of the following three methods.

#### 3.2.1. Gyroscope Integration

The first approach uses the angular rate ω(t) to update the rotation quaternion from sample to sample as shown in  Figure 2b. The quaternion derivative with respect to time is calculated according to [26]:

(3) $$\begin{array}{c} {\dot{q}}_{\omega }\left(t\right)=\frac{1}{2}\;q\left(t-\Delta t\right)⊗{\left[\begin{array}{cccc} 0 & \omega_{x}\left(t\right) & \omega_{y}\left(t\right) & \omega_{z}\left(t\right) \end{array}\right]}^{T} \end{array}$$

This derivative is multiplied with the sampling interval Δt and added to the previous quaternion q(t−Δt). Additionally, normalization is applied and yields the update equation:

(4) $$\begin{array}{c} q\left(t\right)=\frac{q\left(t-\Delta t\right)+{\dot{q}}_{\omega }\left(t\right)\cdot \Delta t}{∥q\left(t-\Delta t\right)+{\dot{q}}_{\omega }\left(t\right)\cdot \Delta t∥} \end{array}$$

#### 3.2.2. Madgwick CF

Madgwick et al. [9] proposed a CF for updating the rotation quaternion based on accelerometer, gyroscope and magnetometer data. Since magnetometer data is not available in our context, the approach is reduced to fusion of accelerometer and gyroscope data. Furthermore, orientation information can only be gained from the accelerometer in static conditions with tolerable degrees of movement. Consequently, we only use the update path from accelerometer data when the magnitude of the acceleration signal $a_{s}\left(t\right)$ in the sensor frame is in certain bounds with respect to gravity in the world frame:

(5) $$\begin{array}{cccc} \left|∥a_{s}\left(t\right)∥-∥g_{w}∥\right| & \leq \gamma & \text{ with}\;g_{w} & =-{\left[\begin{array}{c} 0,1,0 \end{array}\right]}^{T} \end{array}$$

Moreover, all orientation updates from the accelerometer are prevented in the swing phase of the gait cycle. In these high-movement conditions, the orientation update is identical to the gyroscope integration scheme presented earlier. The complete workflow for orientation estimation according to Madgwick is shown in  Figure 2c.

The update equation is identical to Equation (4). However, the purely angular rate-dependent quaternion derivative ${\dot{q}}_{\omega }\left(t\right)$ is replaced by a corrected estimate where the correction term encodes orientation clues gained from the accelerometer:

(6) $$\begin{array}{cccc} \dot{q}\left(t\right) & ={\dot{q}}_{\omega }\left(t\right)-\beta \;\frac{\nabla \epsilon (t)}{∥\nabla \epsilon (t)∥} & \text{ with}\;\nabla \epsilon (t) & =J{\left(\epsilon (t)\right)}^{T}\epsilon \left(t\right) \end{array}$$

where β is a filter parameter proportional to the maximal gyroscope measurement error [9]. The error term ϵ(t) is obtained by transforming gravity in world coordinates $g_{w}$ to sensor coordinates and subtracting the accelerometer measurement in the sensor frame $a_{s}\left(t\right)$. Since quaternions represent sensor-to-world rotations in our case, the transposed rotation matrix yields the inverse transformation:

(7) $$\begin{array}{c} \epsilon \left(t\right)=R{\left(q(t-\Delta t)\right)}^{T}g_{w}-a_{s}\left(t\right) \end{array}$$

The gravity vector $g_{w}$ has only one non-zero element and unit length. Consequently, the multiplication in Equation (7) is identical to selecting a column of the transposed rotation matrix. In the case of gravity being aligned with the *y*-axis, this yields:

(8) $$\begin{array}{c} g_{s}\left(t\right)=R{\left(q(t-\Delta t)\right)}^{T}g_{w}=-\left[\begin{array}{c} 2(q_{1}q_{2}+q_{0}q_{3})\\ q_{0}^{2}-q_{1}^{2}+q_{2}^{2}-q_{3}^{2}\\ 2(q_{2}q_{3}-q_{0}q_{1}) \end{array}\right] \end{array}$$

Additionally, the Jacobian $J\left(\epsilon (t)\right)$ with respect to the quaternion elements needs to be calculated:

(9) $$\begin{array}{c} J\left(\epsilon (t)\right)=J\left(g_{s}\left(t\right)\right)=\;-\left[\begin{array}{cccc} 2q_{3} & 2q_{2} & 2q_{1} & 2q_{0}\\ 2q_{0} & -2q_{1} & 2q_{2} & -2q_{3}\\ -2q_{1} & -2q_{0} & 2q_{3} & 2q_{2} \end{array}\right] \end{array}$$

Finally, the corrected quaternion derivative can be calculated as presented in Equation (6).

#### 3.2.3. Euston CF

Euston et al. [10] presented a CF for orientation estimation of unmanned vehicles. Additionally to gyroscope and accelerometer readings, measurements of airspeed were available. Since such a variable is not of interest in our context, the corresponding path in the block diagram presented in [10] is disconnected. Similar to Madgwick’s orientation filter, orientation updates from accelerometer data are disabled during the swing phase of the gait cycle and only permitted when Equation (5) is valid. The complete workflow is given as a block diagram in  Figure 2d.

For the quaternion update, Equations (3) and (4) are used. However, the measured angular rate ω(t) is replaced by a corrected angular rate signal

(10) $$\begin{array}{c} \omega^{*}\left(t\right)=\omega \left(t\right)+\delta \left(t\right) \end{array}$$

The error term δ is obtained similar to the Madgwick CF. The first step is to convert gravity from world coordinates to sensor coordinates using the previously estimated quaternion. This corresponds to Equation (8). Calculating the cross-product between $g_{s}\left(t\right)$ and the acceleration $a_{s}\left(t\right)$ in the sensor frame, a dimensionless error e(t) is obtained and used in the correction term δ(t):

(11) $$\begin{array}{ccccccccc} e(t) & =\frac{g_{s}\left(t\right)}{∥g_{s}\left(t\right)∥}\times \frac{a_{s}\left(t\right)}{∥a_{s}\left(t\right)∥} & & & & & & \delta (t) & =k_{P}\;e\left(t\right)+k_{I}\int e\left(t\right)\;dt \end{array}$$

The error term e(t) thus describes the angular mismatch between the predicted and measured direction of gravity. The predicted direction $g_{s}\left(t\right)/∥g_{s}\left(t\right)∥$ is based on the tracked transformations between global world and local sensor frame, see Equation (8). The measured direction, however, is directly obtained from the accelerometer reading $a_{s}\left(t\right)$.

In contrast to gyroscope integration or the Madgwick CF, the Euston filter has two adjustable parameters. These are the proportional gain factor $k_{P}$ and the integrator gain factor $k_{I}$. The proportional gain factor is used to separate low-frequency and high-frequency estimates of orientation. For angular rates below $k_{P}$ rad/s, the filter relies on the accelerometer-based estimate. With rising angular rate, the gyroscope data has a higher impact on the estimated orientation. The integrator part of the filter is designed to correct for gyroscope bias.

### 3.3. Coordinate Transform

To represent measured accelerations $a_{s}\left(t\right)$ in the world coordinate frame, the rotation matrices for the computed sequence of quaternions are used:

(12) $$\begin{array}{c} a_{w}\left(t\right)=R\left(q(t)\right)\cdot a_{s}\left(t\right) \end{array}$$

### 3.4. Gravity Removal

Since the acceleration measurement has already been transformed to the world coordinate system, gravity can be subtracted:

(13) $$\begin{array}{c} a\left(t\right)=a_{w}\left(t\right)-g_{w} \end{array}$$

The remainder a(t) is thus the movement component of the measured acceleration signal in the world frame. In order to ensure the cyclic boundary condition $a\left(t_{\mathrm{ms}}\right)=a\left(t_{\mathrm{ms}+1}\right)=0$ that is needed for the re-initialization of the integration step but might have been corrupted during orientation estimation, a dedrifting with a piecewise linear function as explained by Rampp et al. [13] is applied before integration.

### 3.5. Double Integration

The input for this block in the workflow (see  Figure 1a) is the gravity-free acceleration signal in world coordinates a(t). This needs to be integrated twice with respect to time to estimate the foot’s trajectory. Three approaches to accomplish this task are presented in this section. For each stride, the foot’s starting position is initialized in the origin.

#### 3.5.1. Direct Integration

**Figure 3:**
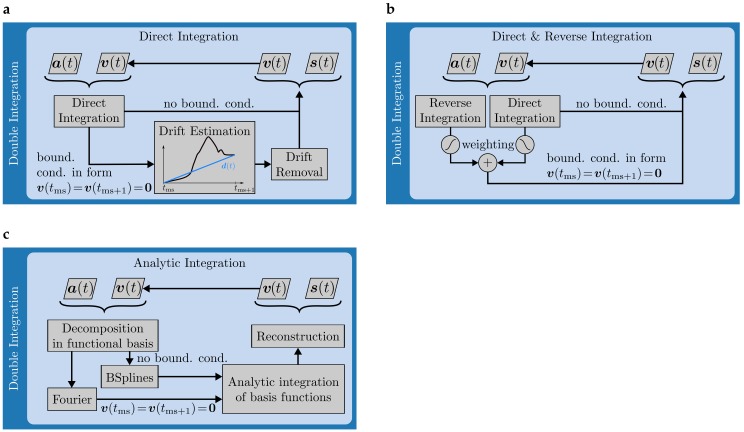
(**a**–**c**) Block diagrams for the double integration methods implemented in the context of this benchmark.

For this approach, the workflow is shown in Figure 3a. It is based on direct integration and linear dedrifting. An estimate for the velocity is obtained via the trapezoidal rule

(14) $$\begin{array}{c} \hat{v}\left(t\right)=\int_{t_{\mathrm{ms}}}^{t}a\left(t\right)\;dt\approx \sum_{i=i_{\mathrm{ms}}+1}^{i_{t}}\frac{a_{i}+a_{i-1}}{2}\cdot \Delta t \end{array}$$

where $i_{\mathrm{ms}}=\left[t_{\mathrm{ms}}/\Delta t\right]$ and $i_{t}=\left[t/\Delta t\right]$ are the corresponding samples in the discrete signal. Due to inaccurate orientation estimation, sensor drift and integration errors, the obtained velocity does not necessarily satisfy the boundary condition $v\left(t_{\mathrm{ms}}\right)=v\left(t_{\mathrm{ms}+1}\right)=0$ (zero-velocity assumption). In order to fulfill this constraint, a linear drift function $d_{v}\left(t\right)$ as described by Rampp et al. [13] or Kitagawa and Ogihara [14] is estimated and subtracted:

(15) $$\begin{array}{c} v\left(t\right)=\hat{v}\left(t\right)-d_{v}\left(t\right) \end{array}$$

The final velocity estimate v(t) is integrated using the trapezoidal rule and the trajectory s(t) is obtained. It is further assumed that the subject walks on level ground, leading to the boundary condition $s_{y}\left(t_{\mathrm{ms}}\right)=s_{y}\left(t_{\mathrm{ms}+1}\right)=0$. To enforce this, the vertical position estimate is dedrifted similarly as already proposed by Kitagawa and Ogihara [14].

#### 3.5.2. Direct and Reverse Integration

Zok et al. [15] proposed to incorporate boundary conditions like the zero-velocity assumption by fusing a direct and a time-reversed integral. The motivation behind this is to use the known initial and final values of an integral as anchor points and compute the integral between these anchors as a weighted sum of both components. The workflow for this method is visualized in  Figure 3b.

In order to obtain a velocity estimate, the direct integral $v_{\to }\left(t\right)$ is computed as shown in Equation  (14). To calculate the time-reversed integral $v_{\leftarrow }\left(t\right)$, the original acceleration signal needs to be flipped with respect to time and direction:

(16) $$\begin{array}{c} a_{\leftarrow }\left(t\right)=-a\left(t_{ms+1}-t\right) \end{array}$$

Equation (14) applied to this signal now yields reverse integral $v_{\leftarrow }^{*}\left(t\right)$. Before fusion, the  transformation $t\to t_{\mathrm{ms}+1}-t$ applied earlier needs to be inverted to ensure common support for both integrals:

(17) $$\begin{array}{c} v_{\leftarrow }\left(t\right)=v_{\leftarrow }^{*}\left(t_{ms+1}-t\right) \end{array}$$

For fusion of both integrals, a sigmoid shaped weighting function w(t)∈[0,1] is used

$$\begin{array}{cccccccccccc} & & & w(t) & =\frac{h\left(t\right)-h\left(t_{ms}\right)}{h\left(t_{ms+1}\right)-h\left(t_{ms}\right)} & & & & & & \text{with}\;h(t) & ={\left(1+\exp\left(-\frac{t-t_{0}}{\eta }\right)\right)}^{-1} \end{array}$$

The shape of w(t) can be influenced by adjusting the steepness parameter η and its mid-point $t_{0}$. The direct and reverse integral are then fused using this weighting function to arrive at the final velocity estimate:

(18) $$\begin{array}{c} v\left(t\right)=\left(1-w(t)\right)\;v_{\to }\left(t\right)+w\left(t\right)\;v_{\leftarrow }\left(t\right) \end{array}$$

The trajectory s(t) is estimated by direct integration of the velocity estimate v(t) in the ground plane and direct and reverse integration in the vertical axis since the level-floor assumption gives rise to a similar boundary condition where the initial and final value of the integral $s_{y}$ are known à priori.

#### 3.5.3. Analytic Integration

Both double integration schemes presented above make use of numerical integration via the trapezoidal rule. As a third integration scheme, Sabatini et al. [17] proposed decomposition of the acceleration signal in a Fourier basis followed by analytic integration of the basis functions and reconstruction of the signal. The theoretical motivation behind this is certainly two-fold: On the one hand, Fourier decomposition ensures a generic implementation of cyclic boundary conditions as the zero-velocity assumption. On the other hand, analytically integrable basis functions promise less integration errors. As an alternative for non-cyclic boundary conditions, we extend the method proposed by Sabatini et al. [17] in the context of this work by decomposition and analytic integration in a B-Spline basis. The workflow for this analytic integration scheme is visualized in  Figure 3c.

Fourier decomposition of the acceleration signal a(t) is given by:

$$\begin{array}{c} \tilde{a}\left(t\right)=c_{0}+\sum_{k=1}^{N_{\mathrm{Fourier}}}c_{1}\left(k\right)\cos\left(\frac{2\pi k}{T}t\right)+c_{2}\left(k\right)\sin\left(\frac{2\pi k}{T}t\right) \end{array}$$

where *T* is the duration of the corresponding stride, $c_{\left\{0,1,2\right\}}$ are the coefficients in the expansion and $\tilde{a}\left(t\right)$ represents the reconstructed signal from the expansion up to order $N_{\mathrm{Fourier}}$. The DC component $c_{0}$ is set to zero to comply with the assumption that we have zero acceleration at the beginning and end of each stride [17]. Time-integration of $\tilde{a}\left(t\right)$ can then be carried out analytically to arrive at estimates for velocity. Likewise, clearance estimates can be obtained by analytical double-integration of the vertical acceleration component ${\tilde{a}}_{y}\left(t\right)$.

For the decomposition and integration in a B-Spline basis, we refer to Dierckx [27]. Relevant parameters regarding this decomposition involve the spline order *k* and the sequence of control points. Both determine the quality with which a signal can be reconstructed once decomposed.

## 4. Experiments

### 4.1. Orientation Estimation

**Figure 4:**
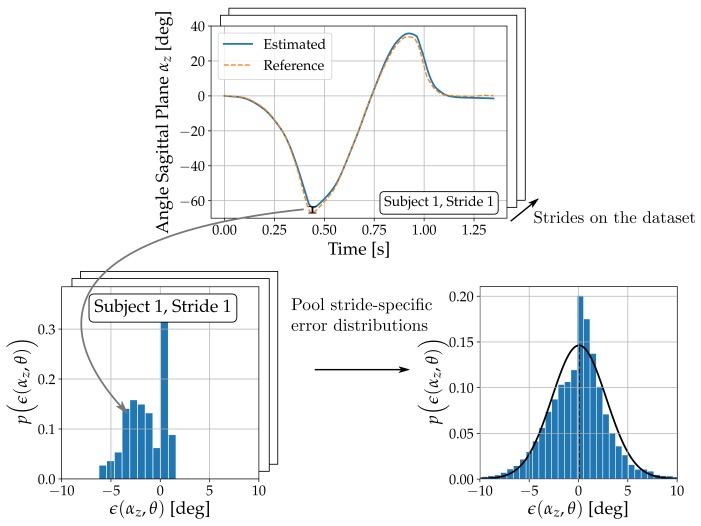
Pooling of individual error distributions yields the error distributions $\epsilon (\alpha_{i},\theta )$ for a given axis i=z, variable and parameter configuration θ.

Suitable parameter sets for the two tunable orientation estimation techniques (Madgwick CF and Euston CF) described above are found via grid-search with reference angle courses obtained from the VICON measurements. For each parameter configuration θ, the error distribution $\epsilon (\alpha_{i},\theta )$ between the sensor-derived and the reference angle courses is computed for each axis i∈{x,y,z} and over all available strides as shown in  Figure 4. The mean error $\frac{1}{3}\sum_{i\in \{x,y,z\}}\mathrm{rmse}\left(\epsilon \left(\alpha_{i},\theta \right)/\rho_{i}\right)$ between the sensor-derived angle courses and the reference angle courses indicates an optimal parameter configuration $\theta^{\mathrm{opt}}$. In this process, error distributions for a given variable $\alpha_{i}$ are normalized by the range $\rho_{i}$ attained on the reference dataset. The parameter grids are shown in  Table 1 and the values for γ are chosen such that the fraction of the stance phase that receives an accelerometer update is sampled equidistantly in [0,1]. For evaluation, the metrics drawn from the error distributions $\epsilon (\alpha_{i},\theta^{\mathrm{opt}})$ are the mean (i.e., accuracy) and its standard deviation (i.e., precision). These allow assessment of estimation errors per axis, provide a comparison between methods and establish a ranking. Additionally, average execution times per stride are monitored.

### 4.2. Double Integration

The three methods for double integration described above mainly differ in the way they address cyclic boundary conditions like the zero-velocity assumption. The direct integration scheme is parameter-less, whereas direct and reverse as well as the analytic integration involve two tunable parameters. For the direct and reverse integration scheme, a grid-search is performed. Only integration endpoints involving such a cyclic boundary condition are considered in order to determine an optimal parameter set θ, in our case v(t) and $s_{y}\left(t\right)$. The evaluation metrics are identical to the ones used to tune the orientation estimation schemes with the difference that the reference time-series for velocity and clearance are used. The resolution of the parameter grid is shown in  Table 1. The analytic integration process, however, involves decomposition of the incoming acceleration signal on a functional basis and the parameters involved determine the reconstruction quality from that decomposition. These parameters are therefore set to ensure average reconstruction errors less than 5% on the complete dataset. For the current application, this corresponds to $N_{\mathrm{Fourier}}=60$ terms. B-Spline decomposition is done with splines of order k=3 while the amount of control points is automatically selected based on a given smoothness goal. This is determined from default settings in the python scipy.interpolate.splrep function, for details see [27].

The optimal parameter set for each method is then evaluated on the complete dataset in order to assess estimation errors for all axes and provide a ranking of methods. Average execution times per stride are measured here as well.

## 5. Results

### 5.1. Orientation Estimation

**Figure 5:**
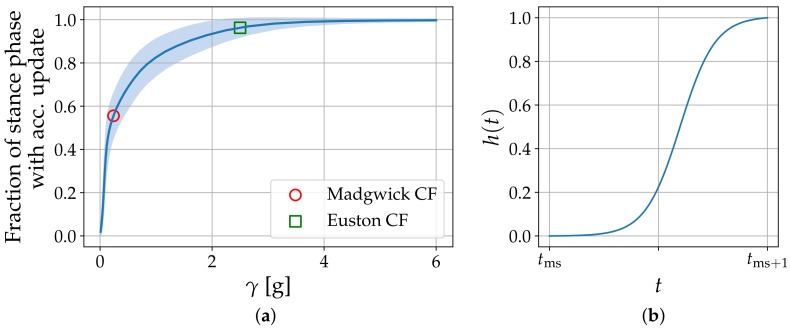
(**a**) Average fraction of the stance phase with an accelerometer orientation update for a given value of γ on the complete dataset. One standard deviation around the mean is shown in light color; (**b**) Optimal weighting function h(t) in the direct and reverse integration scheme for $t_{0}=0.6$ and η=0.08.

Optimal parameter configurations for the two tunable orientation estimation techniques are shown in Table 1. According to the optimal choice for γ, the Euston CF is less sensitive to movement in the fusion of accelerometer and gyroscope orientation estimates compared to the Madgwick CF. Figure 5a gives an overview on the average stance-phase fraction that receives an accelerometer orientation update for a given γ on the current dataset. The optimal operating point for the Madgwick CF thus corresponds to 55% stance-phase coverage while the Euston CF is able to cover 95 % of the stance phase with an orientation update from the accelerometer.

Table 2 shows mean and standard deviation of the corresponding error distributions per plane for all three orientation estimation methods with optimal parameter configurations. Additionally, average execution times per stride for the complete orientation estimation and double integration step are shown. Fusion of gyroscope with accelerometer data as done in the CFs gives slight to no benefits in terms of estimation accuracy or precision over gyroscope integration. Based on these results, a ranking from best to worst performing orientation estimation scheme is given by Madgwick CF → Gyroscope Integration → Euston CF. It is worth noting that the complementary filters need roughly three to five times longer to evaluate on a single stride. In time-critical application scenarios that involve real-time computation, gyroscope integration would therefore be the method of choice as the gain in estimation performance with CFs is only marginal.

### 5.2. Double Integration

Optimal parameter configuration for the direct and reverse integration scheme are listed in Table 1 and Figure 5b shows the corresponding weighting function h(t). Table 3 shows mean and standard deviation of the error distributions for all integration endpoints with a cyclic boundary condition. Results are listed per axis and for all three double integration methods with optimal parameter configurations. For orientation estimation, the Madgwick CF is used in all three cases as this method was ranked best in the previous paragraph. Average execution times per stride for the complete orientation estimation and double integration step are shown as well.

There is large agreement regarding velocity estimation. All methods accomplish this task with comparable accuracy and precision. However, a difference of over 2 cm in precision is observed regarding clearance estimation with direct and reverse integration as compared to the two other methods. A ranking of double integration methods from best to worst performing would therefore be given by Direct and Reverse Integration → Direct Integration → Analytic Integration. Moreover, there is not much overhead in terms of execution time between direct integration and the top ranked method as shown in Table 3.

## 6. Discussion

In order to close a gap in existing literature, three different methods for orientation estimation and double integration in mobile gait analysis have been implemented and evaluated on a common dataset. This resulted in a fair comparison of estimation performance between all methods implemented and the most suitable processing pipeline could be identified.

Regarding orientation estimation, the grid search identified optimal parameter configurations for both CFs involved. In the Madgwick CF, the adjustable parameter β is closely related to the maximal gyroscope measurement error ${\tilde{\omega }}_{\max}=\sqrt{4/3}\;\beta$ (see [9] for details). Our optimal configuration of $\beta^{\mathrm{opt}}=0.046\;\mathrm{rad}/s$ corresponds to a maximal measurement error of $3.04^{\circ }/$s. This order of magnitude seems reasonable given that the gyroscope was not calibrated on a turntable. In the Euston CF, the optimal parameter configuration of $K_{i}^{\mathrm{opt}}=0$ rad/s and $K_{p}^{\mathrm{opt}}=0.0046$ rad/s coincides with the values chosen by Euston et al. [10] who argue that below integration times around 5–10 min, a bias gain of $K_{i}=0$ is a suitable choice due to the slow dynamics involved. For the direct and reverse integration used by Trojaniello et al. [16] in the context of mobile gait analysis, no reference values regarding the weighting function h(t) could be found. However, the optimal shape shown in Figure 5b seems reasonable. The analytic integration scheme needs a Fourier expansion up to order $N_{\mathrm{Fourier}}=60$ in our case which also explains the long execution times. Sabatini et al. [17], however, report expansion up to order 20 with similar reconstruction errors. This is due to a low-pass filter they apply in preprocessing and that is not part of our pipeline. The cut-off frequency of 25 Hz [17] explains the smaller expansion order in their Fourier decomposition.

Regarding the actual estimation performance at optimal parameter configurations, the main difference between orientation tracking methods is observed in the mean error for sagittal plane angles. Here, the Madgwick CF gives a slightly more balanced error distribution compared to the other two methods. This slight improvement, however, comes at a cost of roughly four times longer execution time compared to gyroscope integration. Moreover, gyroscope integration is a parameter-free estimation technique which ensures much broader applicability. In light of the results presented above, the straight-forward gyroscope integration method is as valid as more advanced sensor fusion techniques in the context of single-stride gait analysis.

In the double integration step, differences between methods are more pronounced. Here, a gain of over 2 cm in precision could be achieved for clearance estimation by using the direct and reverse integration scheme over the other two methods. In particular, the analytic integration scheme does not perform better than the direct integration based on numeric integration. Superior performance was one of the theoretical motivations for the analytic integration scheme, but this could not be verified in practice.

The ranking of orientation estimation and double integration methods given above then identifies the most suitable processing pipeline for foot trajectory estimation from inertial data: Madgwick CF for orientation estimation followed by direct and reverse integration for the double integration step. However, some methods in this work are tuned to achieve best possible performance on the given dataset. This of course threatens generalization of the results to other datasets and we do not claim that our dataset is of sufficient size to evade these kind of problems. Nevertheless, orientation estimation with parameter-free gyroscope integration can be achieved at quite similar accuracy and precision compared to the other two methods where the parameter configuration is optimised for the current dataset. Regarding the double integration step, the generalisation of the weighting function h(t) in the top-ranked direct and reverse integration scheme still needs to be shown. However, first results on an independent dataset containing 1220 strides from 101 geriatric patients with the parameter configurations used in this work indicate an identical ranking of double integration methods regarding stride length estimation performance.

For comparison of estimation performance achieved within this work to other works from literature, the sensor clearance trajectory represents a useful measure. Kitagawa and Ogihara [14] report accuracy and precision relative to a motion capture system for three distinct timepoints within the foot clearance trajectory. Based on 180 strides from 10 healthy subjects, they achieved accuracies and precisions of −0.8±5.3 cm, 1.0±5.1 cm and −1.8±3.2 cm for the first and second maximum, as well as the first minimum in the foot clearance trajectory. Mariani et al. [28] provide results of a similar study, but only report absolute accuracies and precisions. For maximum heel clearance, they achieve absolute accuracy and precision of 4.1±2.3 cm based on 378 strides from 12 healthy adults. The top ranked method described in this work reaches sensor clearance estimation with −0.84±3.98 cm regardless of the timepoint within the clearance trajectory which corresponds to an absolute accuracy and precision of 1.97±3.56 cm. These results are well comparable to the studies presented above both in terms of estimation performance as well as number of subjects and study population.

Regarding clinical applications of mobile gait analysis systems, the current work is crucial in determining optimal processing pipelines involved. The clinical use case specifically requires high precision of the measurement technique and complete understanding of the underlying mechanisms. This benchmark identifies optimal processing pipelines based on data from healthy subjects. Acquisition of motion capture referenced patient data and comparison of estimation techniques in patient populations is therefore part of future work.

In summary, we implemented different methods drawn from literature for orientation estimation as well as double integration in the context of mobile gait analysis and evaluated their performance on a common dataset. This closes a gap in existing literature where diverse evaluation datasets make comparison of results difficult. Fusion of accelerometer data with gyroscope signals for orientation estimation gives only slight benefits in performance. Addressing cyclic boundary constraints by fusion of regular and time-reversed integrals, however, increases estimation precision, especially regarding clearance estimation. Contrary to prior assumptions, analytic integration schemes did not outperform methods based on numeric integration.

## Figures and Tables

**Table 1:** Parameter grids for all methods optimized by grid-search on the current dataset and the resulting optimal parameter configuration.

Method	Parameter Grid	Optimal Configuration
Madgwick CF	γ∈{0.04,0.07,0.09,0.14,0.24, 0.45,0.77,1.48,2.50,4.00}	γopt=0.2446
	$\beta \;\;\;=10^{-i/3}\;\;\;i\in \left\{0,1,\cdots ,9\right\}$	βopt=0.0460
Euston CF	γ∈{0.04,0.07,0.09,0.14,0.24, 0.45,0.77,1.48,2.50,4.00}	γopt=2.5046
	$K_{p}=10^{-i/3}\;\;\;i\in \left\{0,1,\cdots ,9\right\}$	$K_{p}^{\mathrm{opt}}=0.0046$
	$K_{i}\;\in \left\{0,\;0.01,\;0.10\right\}$	Kiopt=0.0046
Direct & Reverse Int.	$\begin{array}{rrr} \eta \;\;\;=10^{\kappa -i\cdot \delta } & i & \in \left\{0,1,\cdots ,9\right\}\\ & \kappa & =\log_{10}\left(1/2\right)\\ & \delta & =\frac{\log_{10}\left(1/2\right)+2}{9} \end{array}$	$\eta^{\mathrm{opt}}=0.08$
	$t_{0}\;\;=0.05\cdot i\;\;\;\;\mathrm{with}\;i\in \left\{0,1,\cdots ,20\right\}$	t0opt=0.6046

**Table 2:** Mean ± standard deviation of the error distribution for the estimated angles as well as average execution time for all three orientation estimation schemes with optimal parameter configurations.

	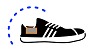	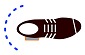	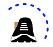	
**Orientation Estimation Scheme**	**Sagittal Plane [deg]**	**Transversal Plane [deg]**	**Frontal Plane [deg]**	**Avg. Exec. Time per Stride [ms]**
Gyro Integration	−0.62±2.94	−2.06±5.29	−1.41±10.82	6.13
Madgwick CF	−0.16±2.93	−2.04±5.24	−1.44±10.83	21.34
Euston CF	−0.62±2.94	−2.06±5.29	−1.41±10.82	31.76

735 strides, 16 healthy subjects.

**Table 3:** Mean ± standard deviation of the error distribution for the estimated velocity/clearance as well as average execution time for all three double integration schemes with optimal parameter configurations and integration endpoints involving cyclic boundary conditions like the zero-velocity assumption.

	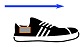	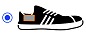	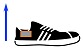	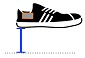	
**Integration Scheme**	**Ant.-Post. Vel. [m/s]**	**Transversal Vel. [m/s]**	**Longitudinal Vel [m/s]**	**Clearance [cm]**	**Avg. Exec. Time per Stride [ms]**
Direct Integration	−0.11±0.37	−0.09±0.69	−0.27±0.44	−0.24±6.66	20.91
Direct & Reverse Int.	−0.09±0.36	−0.09±0.70	−0.23±0.41	−0.84±3.98	22.33
Analytic Integration	−0.10±0.37	−0.09±0.69	−0.28±0.44	−0.32±6.69	101.56

Madgwick CF used for orientation estimation in all three cases. 735 strides, 16 healthy subjects.

**Figure A1:**
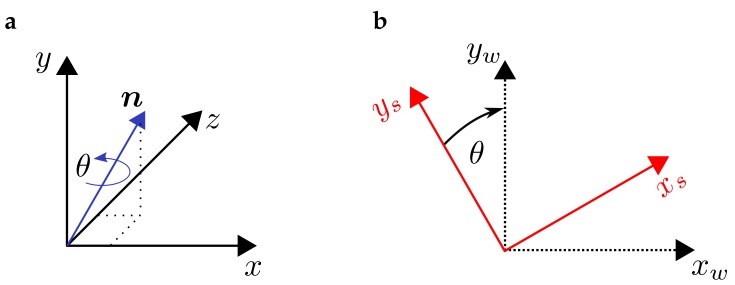
(**a**) 3D-rotation around axis n by angle θ; (**b**) Alignment of two coordinate systems.

## Appendix A. Representation of Rotations

In this work, rotations in three-dimensional space are represented by quaternions. They can be seen as a generalization of how complex numbers efficiently describe rotations in the plane. Because of this ease in notation, quaternions are widely used in applications that depend on efficient treatment of 3D rotations [8,10,11].

A rotation quaternion consists of a real part and three imaginary parts [29,30]. The imaginary units are denoted as *i, j* and *k* and are omitted when using vector notation:

(A1) $$\begin{array}{c} \begin{array}{c} q=q_{0}+q_{1}\cdot i+q_{2}\cdot j+q_{3}\cdot k\equiv {\left[\begin{array}{cccc} q_{0}, & q_{1}, & q_{2}, & q_{3} \end{array}\right]}^{T} \end{array} \end{array}$$

The conjugate of a quaternion is obtained by changing the sign of the imaginary parts. In this work, the conjugate is denoted by a prime:

(A2) $$\begin{array}{c} q^{\prime }={\left[\begin{array}{cccc} q_{0}, & -q_{1}, & -q_{2}, & -q_{3} \end{array}\right]}^{T} \end{array}$$

For quaternions of unit length, the conjugate is identical to the quaternion’s inverse $q^{\prime }=q^{-1}$ [29]. Another way of quaternion notation is to split it into the real part $q_{0}$ and an imaginary vector part $e_{q}$ containing the values from $q_{1}$ to $q_{3}$. Using this notation, the non-commutative product of two quaternions is defined as

(A3) $$\begin{array}{c} p⊗q={\left[\begin{array}{cc} p_{0}q_{0}-e_{p}\cdot e_{q}, & {\left(p_{0}e_{q}+q_{0}e_{p}+e_{p}\times e_{q}\right)}^{T} \end{array}\right]}^{T} \end{array}$$

An axis-angle rotation around an axis n of unit-length and an angle θ, as shown in Figure A1a, can be encoded as a quaternion by:

(A4) $$\begin{array}{c} {}_{w}^{s}q={\left[\begin{array}{cc} \cos(\theta /2), & \sin(\theta /2)\;n \end{array}\right]}^{T} \end{array}$$

where the prescript ${}_{w}^{s}$ denotes that the rotation aligns coordinate system s with the coordinate system w as shown in Figure A1b. Quaternion multiplication can be used to transform a vector from the coordinate system *s* to *w* in the following two ways:

(A5) $$\begin{array}{c} \begin{array}{ccccc} {\left[\begin{array}{cc} 0, & v_{w} \end{array}\right]}^{T} & ={}_{w}^{s}q⊗{\left[\begin{array}{cc} 0, & v_{s} \end{array}\right]}^{T}⊗{}_{w}^{s}q^{\prime } & \mathrm{or} & v_{w} & =R\left({}_{w}^{s}q\right)\cdot v_{s} \end{array}\\ \mathrm{with}\;R\left({}_{w}^{s}q\right)=\left[\begin{array}{ccc} q_{0}^{2}+q_{1}^{2}-q_{2}^{2}-q_{3}^{2} & 2(q_{1}q_{2}-q_{0}q_{3}) & 2(q_{1}q_{3}+q_{0}q_{2})\\ 2(q_{1}q_{2}+q_{0}q_{3}) & q_{0}^{2}-q_{1}^{2}+q_{2}^{2}-q_{3}^{2} & 2(q_{2}q_{3}-q_{0}q_{1})\\ 2(q_{1}q_{3}-q_{0}q_{2}) & 2(q_{2}q_{3}+q_{0}q_{1}) & q_{0}^{2}-q_{1}^{2}-q_{2}^{2}+q_{3}^{2} \end{array}\right] \end{array}$$

Together with (A2), the inverse rotation is obtained by transposing the rotation matrix: $R\left({}_{w}^{s}q^{\prime }\right)=\;R{\left({}_{w}^{s}q\right)}^{T}$. Successive rotations from coordinate system a→b→c can be described efficiently by concatenation of the corresponding transformations

(A6) $$\begin{array}{c} {}_{c}^{a}q={}_{c}^{b}q⊗{}_{b}^{a}q \end{array}$$

The order of concatenation needs special attention here due to the non-commutative nature of the product. Furthermore, the rotation quaternion encodes angles between the corresponding axes in the two 3D coordinate systems. For our application, the axes *x*, *y* and *z* correspond to anterior-posterior, longitudinal and transversal direction. The turning angles around these axes in the frontal, transversal and sagittal plane are given by $\alpha_{x}$, $\alpha_{y}$ and $\alpha_{z}$. They can be calculated as follows:

(A7a) $$\begin{array}{cc} \tan(\alpha_{x}) & =2\left(q_{1}q_{2}+q_{0}q_{3}\right)/\;\left(2\left(q_{0}^{2}+q_{1}^{2}\right)-1)\right) \end{array}$$

(A7b) $$\begin{array}{cc} \sin(\alpha_{y}) & =2(q_{0}q_{2}-q_{1}q_{3})) \end{array}$$

(A7c) $$\begin{array}{cc} \tan(\alpha_{z}) & =2\left(q_{2}q_{3}+q_{0}q_{1}\right)/\;\left(2\left(q_{0}^{2}+q_{3}^{2}\right)-1)\right) \end{array}$$
